# Ultrahigh Frequency (100 MHz–300 MHz) Ultrasonic Transducers for Optical Resolution Medical Imagining

**DOI:** 10.1038/srep28360

**Published:** 2016-06-22

**Authors:** Chunlong Fei, Chi Tat Chiu, Xiaoyang Chen, Zeyu Chen, Jianguo Ma, Benpeng Zhu, K. Kirk Shung, Qifa Zhou

**Affiliations:** 1 School of Microelectronics, Xidian University, Xi’an 710071, China; 2 NIH Resource Center for Medical Ultrasonic Transducer Technology and Department of Biomedical Engineering, University of Southern California, Los Angeles, California 90089, USA

## Abstract

High resolution ultrasonic imaging requires high frequency wide band ultrasonic transducers, which produce short pulses and highly focused beam. However, currently the frequency of ultrasonic transducers is limited to below 100 MHz, mainly because of the challenge in precise control of fabrication parameters. This paper reports the design, fabrication, and characterization of sensitive broadband lithium niobate (LiNbO_3_) single element ultrasonic transducers in the range of 100–300 MHz, as well as their applications in high resolution imaging. All transducers were built for an *f-number* close to 1.0, which was achieved by press-focusing the piezoelectric layer into a spherical curvature. Characterization results demonstrated their high sensitivity and a −6 dB bandwidth greater than 40%. Resolutions better than 6.4 μm in the lateral direction and 6.2 μm in the axial direction were achieved by scanning a 4 μm tungsten wire target. Ultrasonic biomicroscopy images of zebrafish eyes were obtained with these transducers which demonstrate the feasibility of high resolution imaging with a performance comparable to optical resolution.

As one of the most important and well established tools, ultrasound imaging provides noninvasive valuable diagnostic information, especially in the form of cross-sectional images of soft tissues^1^,^2^,^3^,^4^. Conventional ultrasonic imaging system typically works at frequencies below 100 MHz, which provides tens of microns to millimeter spatial resolution^5^,^6^,^7^. High resolution clinical imaging has shown promise in morphological studies of the living corneal epithelium, in visualizing epidermis, in pediatrics as well as in small animal imaging for drug and gene therapy. Visualization of the corneal epithelium provides insight into corneal diseases and the effects of photorefractive surgery, also visualization of the epidermis could permit early diagnosis of melanoma^8^,^9^. Nevertheless, one of reasons that have prevented ultrasonic imaging resolution from further improving has been the unavailability of highly sensitive wide band ultrasound transducers at frequencies higher than 100 MHz. Because of the required need in precisely controlling fabrication parameters of a high frequency ultrasound transducer, transducers at ultrahigh frequencies (>200 MHz) for ultrasound imaging has rarely been reported to the best of the authors’ knowledge.

To improve spatial resolution, one strategy is to increase the operating frequencies accompanied by a loss of penetration^10^,^11^. The axial resolution is determined by the pulse duration or the bandwidth of the pulse. The lateral resolution at the focal point is determined by the product of wavelength and the *f-number*, (the ratio of the focal distance to the transducer aperture). For a fixed number of cycles per pulse, an increase in frequency would result in a reduction in wavelength and thus pulse length. One challenge of developing such high frequency ultrasound imaging system is the high attenuation (∝*f* in tissues) of high frequency ultrasonic waves^12^. To achieve high lateral resolution and adequate sensitivity, a highly focused, low *f-number* transducer design was implemented. Hence, the high frequency, low *f-number* imaging yields improved spatial resolution at the focal point at the expense of imaging depth^1^,^9^,^13^.

In this paper, we report the design, fabrication, and characterization of press-focused LiNbO_3_ transducers at frequencies of 100–300 MHz. The high resolution imaging capability of the transducers was demonstrated by scanning a zebrafish eye. The fine structures of the eye were discernible with these ultrahigh frequency transducers.

## Results and Discussion

### Transducer Morphology

**Figure 1:**
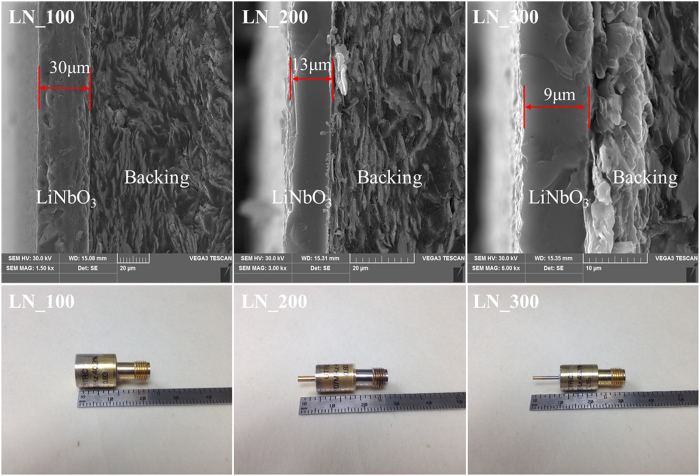
Cross sections of LiNbO_3_ piezoelectric elements of different thicknesses imaged by SEM (above figures), and photographs of finished devices (below figures).

In this work, three kinds of transducers with center frequencies of 100 MHz, 200 MHz and 300 MHz were fabricated and labelled as “LN_100”, “LN_200” and “LN_300”. The “LN_100” ultrasonic transducer was fabricated using conventional technology^3^. The “LN_200” and “LN_300” transducers were designed and fabricated as needle type transducers due to their minute element size. Scanning electron microscope (SEM, JSM-6700, JEOL, Peabody, MA) validated that the average thickness of piezoelectric element of the transducers was 29 μm, 13 μm and 9 μm, which was labeled as “LN_100”, “LN_200”, and “LN_300” in Fig. 1. The photographs of the completed press-focused ultrahigh frequency LiNbO_3_ transducers were presented at bottom of Fig. 1.

### Transducer Characterization

**Figure 2:**
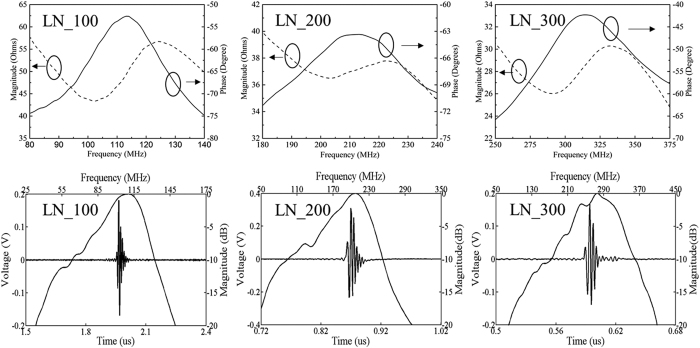
Electrical impedance magnitude and phase plots for the water-resonating LiNbO_3_ transducers (above figures); and time-domain pulse/echo response and normalized frequency spectrum of LiNbO_3_ transducers (below figures).

The frequency dependence of the electrical impedance (both magnitude and phase) of the transducers is displayed in Fig. 2, which was measured by Agilent 4991A impedance analyzer (Agilent Technologies, Santa Clara, CA). The resonant frequency (*f*_*0*_) and corresponding impedance (*Z*) were determined from the plots. The electromechanical coupling coefficient (*k*_*t*_) was calculated as follows:


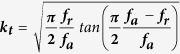


where *f*_*r*_ and *f*_*a*_ denote resonant and antiresonant frequency, respectively. The parameters were list in Table 1.

**Figure 3:**
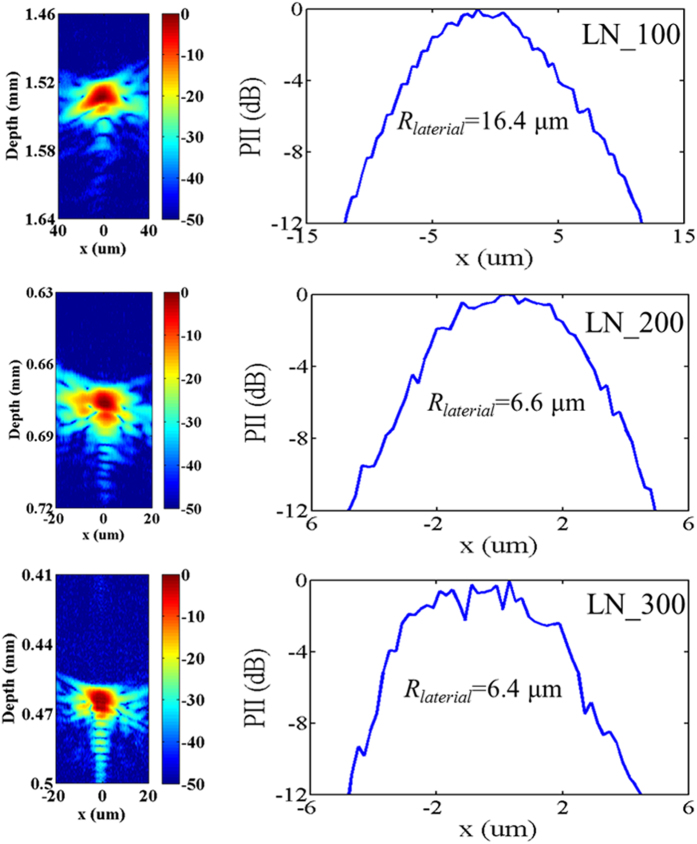
Image of 4 μm tungsten wire target (left figures); (b) Lateral beam profile of the LiNbO_3_ transducers (right figures).

The conventional pulse-echo response measurement was carried out in distilled water. The transducer was connected to a JSR Ultrasonics DPR 500 (Imaginant, Pittsford, NY) pulser/receiver and excited by an electrical impulse at 200 Hz repetition rate and 50 Ω damping. The energy involved was 2.3 μJ and no gain was applied in excitation or reception. An X-cut quartz plate was placed at the focal point as a target. The receive-echo response and frequency spectra of the LiNbO_3_ press-focused transducer are shown in Fig. 3. The measured transducer performance was list in Table 2.

For transducers “LN_100” and “LN_200”, the measured center frequencies were close to the impedance resonant frequencies, while for transducer “LN_300”, the measured center frequency at focus point was 275 MHz, lower than the simulated resonant frequency 315 MHz. A possible cause of the frequency down-shift is the frequency dependent attenuation in water, as the attenuation of ultrasound is proportional to the square of the frequency. All the transducers show relatively broad −6 dB bandwidth (>40%). The small *f-number* which leads to narrow beam width is expected to yield high resolution imaging.

Meanwhile, the lateral beam profile of the transducers was evaluated by scanning a tungsten wire target (California Fine Wire, Grover Beach, CA) with 4 μm diameter. The pulse intensity integral (PII) was calculated from the wire target. As shown in Fig. 3, a beam width equal to 16.4 μm, 6.6 μm, and 6.4 μm was obtained by transducers “LN_100”, “LN_200” and “LN_300” in detecting a spatial point target at full width at half maximum (FWHM, −6 dB), which agree with the theoretical values of 12.4 μm, 6.2 μm and 6.4 μm (= *f-number* × wave length at center frequency). In addition, the axial resolution was calculated from the FWHM of the pulse length of the echo, which was 15.4 μm, 8.5 μm and 6.2 μm, respectively.

### High Resolution Zebrafish Eye Imaging

These LiNbO_3_ transducers were used to image the anterior portion of the zebrafish eye by using a customized ultrasonic biomicroscopy (UBM) system. A logarithmic compression algorithm was used to improve grayscale visualization of the image. Each 2-D frame of image data was obtained by scanning the transducers and collecting pulse-echo lines at 2 μm spacing. The imaging depth was determined by the ultrasound penetration depth.

**Figure 4:**
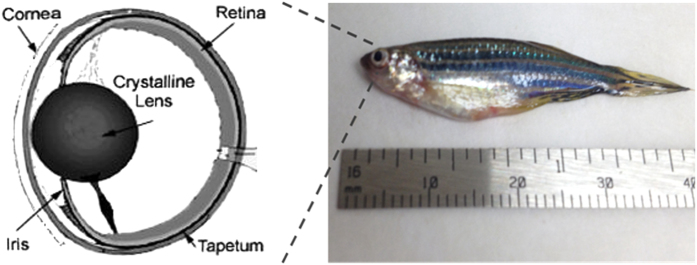
Photograph of fish specimen and its eye structure diagram.

The fish specimen as well as the structure diagram of its eye was presented in Fig. 4. All zebrafish studies were performed in compliance with IACUC protocol No. 10985 approved by USC. The whole length of the specimen is around 40 mm, and the diameter of its eye is around 2 mm. The crystalline lens of a zebrafish’s eye is purely spherical. Unlike mammals who use special muscles to change the shape of lens, the fish moves the lens backwards and forwards to adjust the focus. In such situation, the distance between lower cornea surface and lens surface could be quite close.

**Figure 5:**
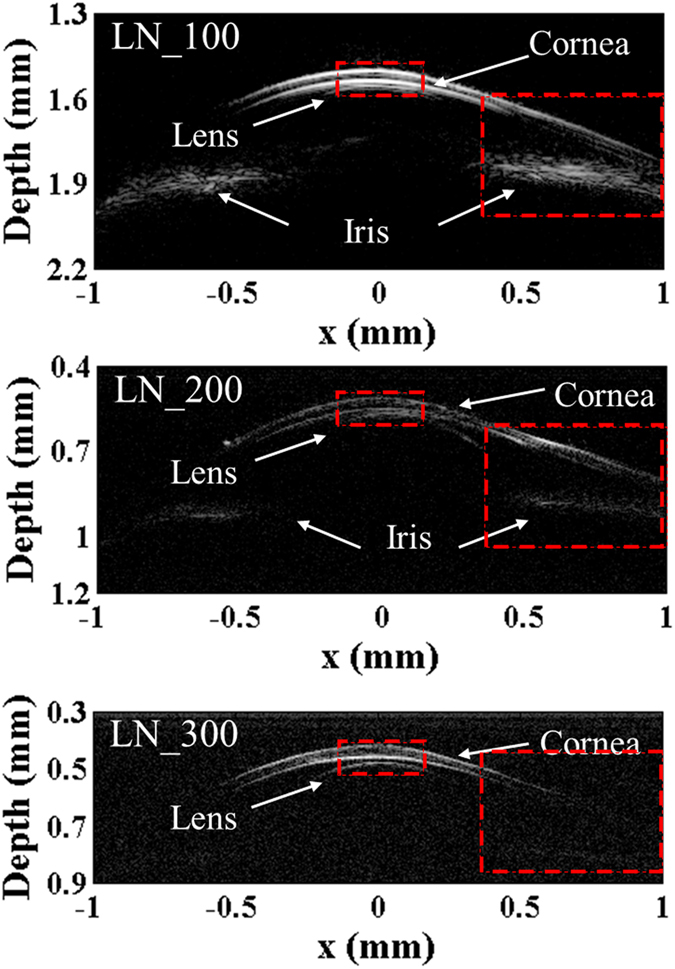
UBM images of the anterior portion of a zebrafish eye.

**Figure 6:**
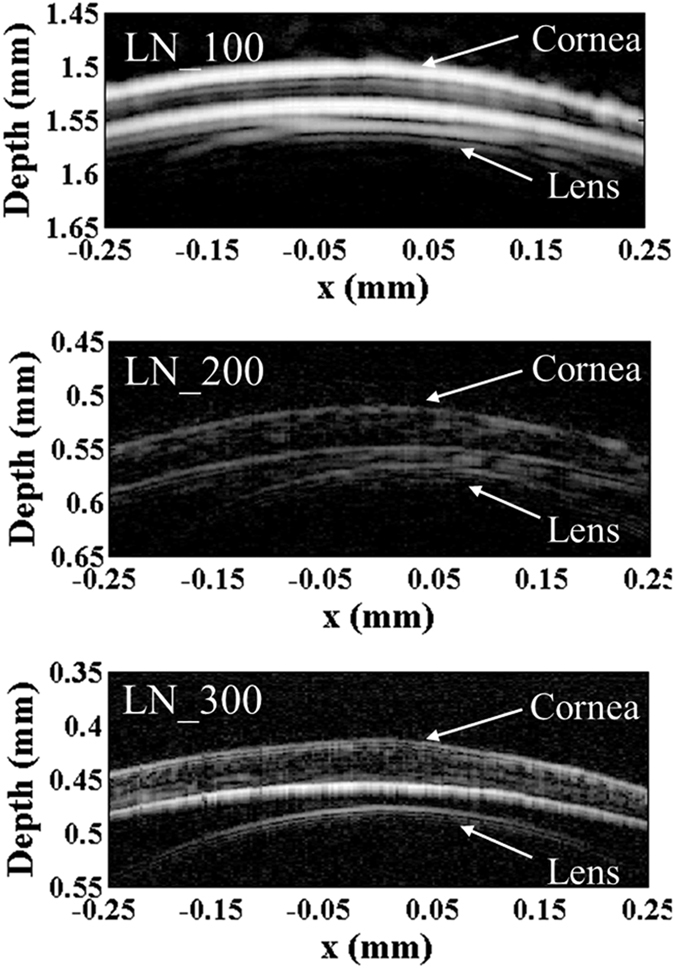
Enlarged view of the center of cornea.

The images of fish eye (Fig. 5) displayed high resolution and good signal-to-noise ratio. The image obtained by the transducer “LN_100”, exhibited clear structure of cornea, lens surface and iris. With higher operating frequency, the resolution improved while the imaging depth decreases. The larger view of the red box marked in Fig. 5 was presented in Fig. 6. According to the images, the thickness of the cornea of the specimen is around 50 μm, and the distance between lower surface of cornea and lens surface is around 10 μm.

**Figure 7:**
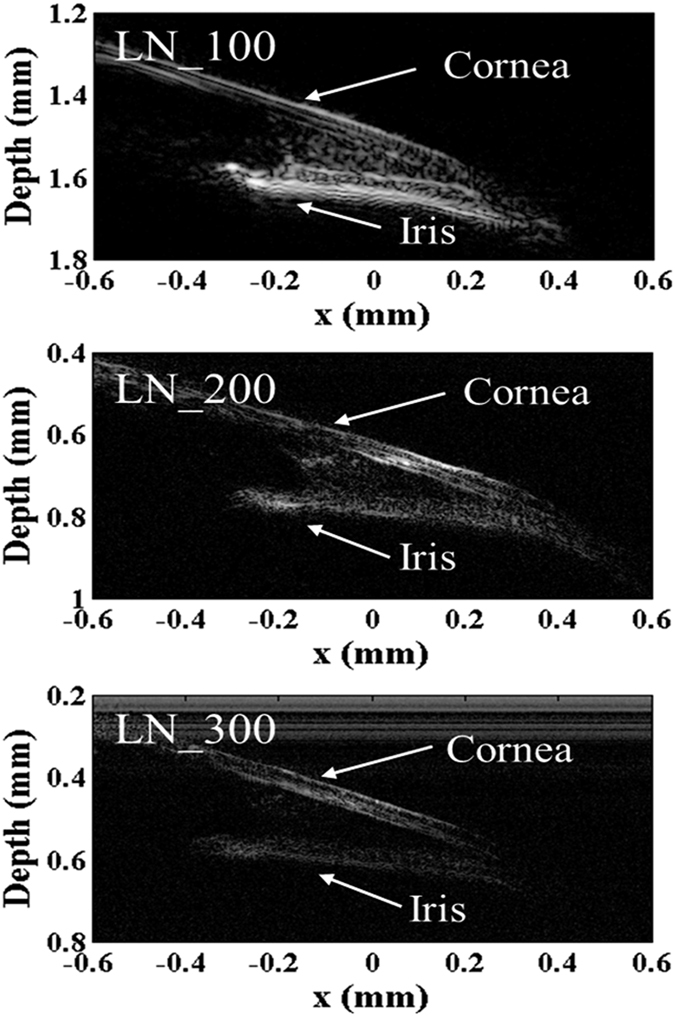
UBM images of the corner of a zebrafish eye.

Figure 7 shows the detail information of the corner of eye. The cornea and the iris as well as the space structure between them are clear demonstrated. Significant finer details were resolved at 300 MHz versus the ones at lower frequencies.

## Conclusion

This study reports the design, fabrication, and characterization of LiNbO_3_ ultrahigh frequency, broadband, high sensitivity and small *f-number* transducers. The performance of these transducers was in good agreement with the simulation in terms of center frequency, bandwidth and beam width. Tungsten wire imaging of the transducers showed a lateral resolution of 6.4 μm and an axial resolution of 6.2 μm, which are comparable to optical imaging resolution. The application of these transducers in high resolution imaging was demonstrated via the image of zebrafish eyes. These high resolution UBM images illustrate that availability of ultrahigh frequency transducers will make UBM a promising tool to study fine biological structures.

## Materials and Methods

In this work, 36° Y cut Lithium niobate single crystal was selected because it exhibits good electromechanical coupling (kt ~ 49%), low dielectric permittivity (*ε*^s^ − 39), high longitudinal wave velocity (~7340 m/s) and high Curie temperature (~1150 °C).

Specific design parameters of the transducers, including the aperture size and proper thickness of acoustic stacks were optimized through a Krimboltz, Leedom, and Mattaei (KLM)^14^ model-based simulation software PiezoCAD (Sonic Concepts, Woodinville, WA). Material properties used for transducer design consideration and the optimized design specifications are listed in Tables 3 and 4.

**Figure 8:**
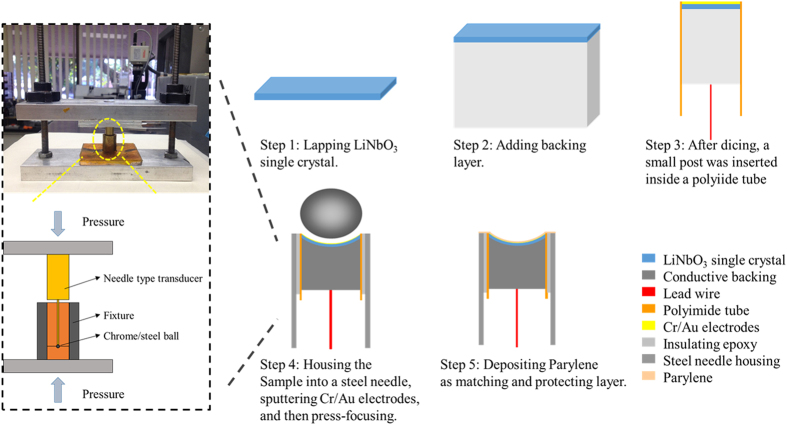
The schematic fabrication flow of press-focused LiNbO_3_ needle transducer.

Figure 8 shows a schematic fabrication flow of the press-focused LiNbO_3_ needle transducer. “LN_200” and “LN_300” transducers had similar fabrication process, which was presented in detail using “LN_300” as an example.

1. The 36° rotated Y-cut LiNbO_3_ single crystal (Boston Piezo-optics, Bellingham, MA) was manually lapped to around 10 μm according to the design and simulation. This lapping process was carried out with extreme care and in a patient manner.
2. Cr/Au (50/100 nm) electrodes were sputtered on one side of the LiNbO_3_ and E-solder 3022 was then cast on this side as the backing material, which was lapped to 2 mm.
3. The sample was diced to 0.4 × 0.4 mm^2^ posts using a dicing saw (Tcar 864-1, Thermocarbon, Casselberry, FL), and then housed inside a polyimide tube with an inner diameter of 0.57 mm. A lead wire was connected to the backing layer with an additional amount of conductive epoxy. The polyimide tube provided electrical isolation from the needle housing.
4. The transducer was housed in a subminiature version A (SMA) connector. A layer of Cr/Au (50 nm/100 nm) was sputtered across the transducer front surface to form the ground plane connection.
5. The device was then press focused by a highly polished chrome/steel ball bearings (0.8 mm diameter, grade 3, Bal-Tec, Los Angeles, CA) at 90 °C. A set of customer design fixture was utilized to accomplish press-focusing.
6. Finally, a thin layer of parylene (~1.5 μm) was vapor-deposited by a PDS 2010 Labcoator (Specialty Coating Systems, Indianapoils, IN) on the front face of the transducer, serving as an acoustic matching layer and a protection layer.

## Additional Information

**How to cite this article**: Fei, C. *et al.* Ultrahigh Frequency (100 MHz–300 MHz) Ultrasonic Transducers for Optical Resolution Medical Imagining. *Sci. Rep.* **6**, 28360; doi: 10.1038/srep28360 (2016).

## Figures and Tables

**Table 1:** Electrical impedance specifications of the transducers.

	LN_100	LN_200	LN_300
*f*_*0*_ (MHz)	113	212	315
*Z* (Ω)	50	38	29
*f*_*r*_ (MHz)	103	200	291
*f*_*a*_ (MHz)	125	222	333
*k*_*t*_	0.44	0.47	0.52

**Table 2:** Measured transducer performance.

	LN_100	LN_200	LN_300
Center frequency (MHz)	104	207	275
Peak to peak Voltage @0 dB gain (mV)	355	700	280
−6 dB Bandwidth	40.3	44.2	45.05
−6 dB Pulse length (nsec)	20	12	8
Focus depth (mm)	1.52	0.66	0.46
*f-number*	0.84	0.83	1.15

**Table 3:** Passive material properties used in the transducer designs^1^.

Material	Function	*c* (m/s)	*ρ* (kg/m^3^)	*Z* (MRayl)
LiNbO_3_	Piezoelectric element	7360	4688	34.5
Parylene	Matching layer	2350	1100	2.58
Water	Front load	1540	1000	1.54
E-Solder 3022	Conductive backing	1850	3200	5.92
EPO-TEK 301	Insulating epoxy	2650	1150	3.05

^1^*c* is the longitudinal sound velocity; *ρ* is the density; *Z* is the acoustic impedance.

**Table 4:** Design specifications of LiNbO_3_ transducers.

Specification	LN_100	LN_200	LN_300
Center frequency (MHz)	100	200	300
Thickness of LiNbO_3_ (μm)	29	15	10
Thickness of Parylene (μm)	5	2.5	1.5
Aperture size	ϕ1.8 mm	0.8 × 0.8 mm^2^	0.4 × 0.4 mm^2^
